# Between catatonia and neuroleptic malignant syndrome: the thin line of a diagnostic dilemma

**DOI:** 10.1192/j.eurpsy.2026.12171

**Published:** 2026-07-31

**Authors:** C. Garcia Cerdan, J. I. de la Iglesia Larrad, M. Ligero Argudo, I. M. Peso Navarro, R. K. González Bolaños, P. Salas Aranda, A. Sánchez Chillón, E. Domínguez Álvarez, P. Andrés Olivera

**Affiliations:** 1 Psychiatry, University Clinical Hospital of Salamanca, Salamanca; 2 Psychiatry, General Hospital of Segovia, Segovia, Spain

## Abstract

**Introduction:**

Catatonia is a complex psychomotor syndrome that can occur in a variety of psychiatric and medical conditions. Neuroleptic malignant syndrome (NMS) shares clinical features with catatonia, such as rigidity, mutism, altered consciousness, and fever, making differential diagnosis difficult. Distinguishing between the two is crucial, as management and prognosis differ significantly.

**Objectives:**

To present a complex clinical case with overlapping features of catatonia and NMS, highlighting diagnostic challenges and the importance of a multidisciplinary approach.

**Methods:**

Descriptive analysis of a case admitted to a psychiatric inpatient unit, complemented by clinical evaluation, laboratory and imaging tests, and a review of the relevant literature.

**Results:**

A 62-year-old male with paranoid schizophrenia on long-term treatment with clozapine and paliperidone was admitted due to mutism, refusal to eat, generalized rigidity, and confusional syndrome. Given the suspicion of NMS, antipsychotics were discontinued, and benzodiazepines plus medical support were initiated.

During hospitalization, he did not develop fever or laboratory abnormalities compatible with NMS, and the clinical picture was reoriented toward catatonia. After complete withdrawal of antipsychotics, there was an abrupt reappearance of delusional symptoms with marked anguish, raising doubts about whether this was part of the confusional state or a new psychotic decompensation. Once laboratory tests normalized and no vital risk remained, the patient was transferred to the psychiatry ward, where paliperidone palmitate was progressively reintroduced, achieving clinical stabilization, partial remission of psychotic symptoms, and gradual improvement of rigidity and psychomotor inhibition.

**Conclusions:**

This case illustrates how catatonia may closely mimic neuroleptic malignant syndrome, generating significant diagnostic uncertainty in patients on long-term antipsychotic treatment. In this situation, the absence of fever and relevant laboratory abnormalities was crucial to rule out NMS. The complete withdrawal of antipsychotics, although necessary during the acute phase, unmasked the patient’s underlying psychotic vulnerability, further complicating the clinical course. Ultimately, the gradual reintroduction of antipsychotic treatment facilitated partial remission of psychotic symptoms as well as progressive improvement of rigidity and psychomotor inhibition. These findings emphasize the importance of a flexible, stepwise, and multidisciplinary approach in managing such complex cases.

**Disclosure of Interest:**

None Declared

